# Pandemic-specific coping, anxiety, and depression across multiple waves of COVID-19 in elite athletes with disabilities

**DOI:** 10.3389/fpsyg.2023.1256853

**Published:** 2023-10-11

**Authors:** Piotr K. Urbański, Tomasz Tasiemski, Britton W. Brewer

**Affiliations:** ^1^Department of Adapted Physical Activity, Poznań University of Physical Education, Poznan, Poland; ^2^Department of Psychology, Springfield College, Springfield, MA, United States

**Keywords:** coping styles, mental health, paralympic, COVID-19, anxiety, depression

## Abstract

**Objectives:**

Competitive athletes have faced many of the same mental health challenges experienced by the general population during the COVID-19 pandemic. The purpose of the current study was to examine the extent to which pandemic-specific coping predicted anxiety and depression over and above general coping styles in elite athletes with disabilities across multiple waves of the COVID-19 pandemic.

**Methods:**

Participants were 91 athletes (60 men and 31 women) in the Polish Paralympic Preparation Program before the 2020 Tokyo Summer Paralympic Games and 2022 Beijing Winter Paralympic Games. The Coping Inventory for Stressful Situations and an open-ended item asking participants to describe coping resources they had used to overcome stressful situations caused by the pandemic were administered in April 2021, and the Hospital Anxiety and Depression Scale was administered in April, July, and November of 2021.

**Results:**

General coping styles were not significantly associated with pandemic-specific coping, anxiety, or depression in the July and November 2021 assessments. Pandemic-specific coping was related prospectively to both anxiety and depression across the July and November 2021 assessments when controlling for age, gender, general coping styles, and April 2021 anxiety and depression, respectively.

**Conclusion:**

The findings suggest that elite athletes with disabilities may cope with pandemic-related stress differently from how they cope with stress in general and that pandemic-specific coping may be relevant to mental health outcomes during the COVID-19 pandemic. This information may be useful in the development of interventions to assist elite athletes with disabilities cope with pandemics and other atypical stressors.

## Introduction

In March 2020, the WHO (2020) declared the disease caused by the SARS-CoV-2 novel coronavirus (i.e., COVID-19) a global pandemic. Nations around the world implemented measures such as social distancing, lockdowns, quarantines, and travel restrictions to reduce the spread of infection. The COVID-19 pandemic and attempts to mitigate its effects had a rapid and dramatic adverse impact on social, economic, and healthcare systems worldwide (Nicola et al., 2020), producing a vast array of stressors for the public (Xiong et al., 2020; Ciciurkaite et al., 2022).

The COVID-19 pandemic has presented a multitude of challenges for various segments of the population, such as athletes with disabilities, including Paralympic athletes. Research has suggested that athletes with disabilities may be at an increased risk of contracting the virus, especially if their disabilities pertain to respiratory or immune system issues, or involve visual and motor impairments (International Paralympic Committee, 2020; Akashi et al., 2022; Muti et al., 2022). Once infected, the effects of COVID-19 or lockdown measures on these athletes with disabilities can be complex. Beyond the direct health consequences of COVID-19, there can be negative effects on anxiety levels, sleep patterns, eating habits, and other areas of daily functioning (Hall et al., 2021; Taheri et al., 2023a,b). During the COVID-19 pandemic, in addition to the challenges faced by the general population, athletes in general and athletes with disabilities in particular have had to contend with sport-specific stressors. Among these stressors are reduced interactions with coaches and teammates, cancelations or postponements of competitions, and limited access to training facilities. Naturally, these effects have led to decreased physical activity, diminished training volume, and deteriorated physical fitness, which in turn have affected return-to-play decisions and heightened the risk of injury (Hu et al., 2021; Urbański et al., 2021; Vincent et al., 2022). As a consequence, there have been significant setbacks in performance continuity and readiness, resulting in a regression of previously acquired skill levels (Cavaggioni et al., 2022; Puce et al., 2022). In contrast, Shaw et al. (2021) reported no negative impact of COVID-19 on para-athlete training, suggesting that the pandemic did not interfere with training activities, and other studies’ investigators have observed that Paralympic athletes actually had a positive reaction to the COVID-19 situation (Clemente-Suárez et al., 2020; Martínez-Patiño et al., 2021). Potential explanations for these discrepant findings include variations in the type and specificity of sports practiced by study participants, use of cross-sectional research designs, timing of data collection in relation to the corresponding phase of the pandemic, and small sample sizes of some of the studies (Puce et al., 2022).

Accompanying the disruptive and stress-inducing effects of the COVID-19 pandemic was a decline in the mental health of the general population marked by increases in anxiety and depression (Vindegaard and Benros, 2020; Xiong et al., 2020). Similar deleterious effects of the pandemic on mental health have been documented in athletes (Denerel and Lima, 2021; Jia et al., 2022; Vincent et al., 2022), especially high-level (i.e., elite) athletes (Jia et al., 2022). A comprehensive meta-analysis by Puce et al. (2022) highlighted numerous areas in which athletes with disabilities have been affected by COVID-19. One factor that may help athletes to mitigate the impact of the COVID-19 pandemic on mental health is coping, which refers to the cognitive and behavioral attempts that people make to manage the demands of situations they perceive as stressful (Lazarus and Folkman, 1984). Coping efforts initiated by athletes in response to the pandemic include adjusting their goals (Costa et al., 2022), engaging in sport training, doing other activities, talking with their coaches (and others), and keeping a positive mindset (Bezzina et al., 2021; Hong and Allen, 2022). Findings from quantitative studies have shown that self-reported use of coping strategies such as receiving social support, maintaining social connections (Graupensperger et al., 2020), cognitive restructuring, keeping emotionally calm (Leguizamo et al., 2021), and mindfulness (Myall et al., 2021) are positively associated with mental health outcomes during the COVID-19 pandemic.

Pété et al. (2022) adopted a person-centered approach to investigate athletes’ preferred combination of strategies to cope with the COVID-19 pandemic. Four coping profiles were identified: (a) active and social, (b) avoidant, (c) engaged, and (d) self-reliant. Athletes with the active and social profile reported high levels of cognitive restructuring, distraction, and problem solving. Athletes with the avoidant profile endorsed the use of avoidant strategies such as denial, disengagement, and substance use. Athletes with the engaged profile expressed a preference for problem solving and cognitive restructuring. Athletes with the self-reliant profile reported moderate levels of distraction and cognitive restructuring. The highest level of anxiety was reported by athletes with the avoidant coping profile.

As noted by Pété et al. (2022), athletes have faced “unprecedented and unknown situations with increased risk of exposure to multiple stressors” (p. 238) during the COVID-19 pandemic. The health risks and inequitable effects of pandemic-related restrictions have been especially pronounced among elite athletes with disabilities (Bundon et al., 2022), who have been included in relatively few studies of athlete mental health during the COVID-19 pandemic (Jia et al., 2022). Although athletes with disabilities reported less distress during the pandemic than able-bodied athletes in one study (Fiorilli et al., 2021), persons with disabilities reported experiencing greater pandemic-related stress than persons without disabilities in the general population, and Paralympic athletes reported lower levels of mental health than a sample matched on age and gender from the general population (Busch et al., 2022) in other studies. Moreover, given the uniqueness of the circumstances, it is possible that the item content on standardized coping inventories may not fully capture the range of coping activities with which people engage and that responses to such inventories may not reflect what people do when confronted by the set of stressors characteristic of the COVID-19 pandemic (Urbański et al., 2023). It is, therefore, necessary to consider pandemic-specific coping efforts when investigating the association between coping and mental health outcomes during the pandemic. Consequently, the purpose of the current study was to examine prospectively and for the first time the extent to which pandemic-specific coping was related to anxiety and depression in elite athletes with disabilities when statistically controlling for more general coping tendencies (i.e., avoidance-, emotion-, and task-oriented coping) over multiple assessments.

## Methods

### Participants

Participants were 91 individuals (60 men and 31 women) enrolled in the Polish Paralympic Preparation Program in advance of the 2020 Tokyo Summer Paralympic Games (held in August and September 2021) and 2022 Beijing Winter Paralympic Games (held in March 2022). Participants reported a wide variety of disabilities: amputation (*n* = 26, 29%), spinal cord injury (*n* = 20, 22%), visual impairment (*n* = 16, 18%), cerebral palsy (*n* = 6, 7%), muscular dystrophy (*n* = 1, 1%), and other (*n* = 22, 24%). Participants represented a wide variety of sports as well, with swimming (*n* = 15, 17%), athletics (*n* = 14, 15%), goalball (*n* = 12, 13%), cycling (*n* = 11, 12%), and fencing (*n* = 10, 11%) reported most frequently. Participants reported a mean age of 31.02 (*SD* = 12.04) years, a mean of 17.85 (*SD* = 14.48) years since the occurrence of their injury or diagnosis of disease, and a mean of 9.56 (*SD* = 7.92) years of Paralympic experience.

### Measures

As part of a larger study of psychological responses to the COVID-19 pandemic, participants completed measures of demographic and sport-related variables, coping, anxiety, and depression. Demographic and sport-related variables assessed via a self-report questionnaire included age, gender, disability, duration of disability, sport, Paralympic experience, actual hours of training, and intended hours of training.

Coping with stressors in general was assessed with the Polish version (Strelau et al., 2005) of the Coping Inventory for Stressful Situations (CISS; Endler and Parker, 1994). Responses to the 48 items on the CISS are given on a 5-point Likert-type scale from 1 (*not at all*) to 5 (*very much*). The CISS has subscales measuring avoidance-oriented coping (AO), emotion-oriented coping (EO), and task-oriented coping (TO) over the prior month. Strelau et al. presented support for the reliability and validity of the Polish version of the CISS, including Cronbach’s alpha coefficients ranging from 0.74 to 0.88. In the current study, Cronbach’s alpha coefficients of 0.88, 0.92, and 0.92 were obtained for the AO, EO, and TO subscales, respectively. The Polish version of the CISS was used to assess coping during the COVID-19 pandemic in a previous study (Rogowska et al., 2021). To assess coping specific to the COVID-19 pandemic, participants were asked to describe coping resources they had used to overcome stressful situations caused by the pandemic. An open-ended item was used due to the novelty of the pandemic situation and the lack of an appropriate extant measure of the construct for athletes with disabilities.

Anxiety and depression were assessed with the Hospital Anxiety and Depression Scale (HADS; Zigmond and Snaith, 1983), which has frequently been used for this purpose in samples of persons with disabilities (Woolrich et al., 2006). The HADS consists of two 7-item subscales measuring anxiety (HADS-A) and depression (HADS-D), respectively. Responses to HADS items are given on a scale from 0 (*very rarely*) to 3 (*often*). Thus, total scores on the HADS-A and HADS-D range from 0 to 21. Higher scores on the HADS-A and HADS-D correspond with higher levels of anxiety and depression, respectively. Cronbach’s alpha coefficients in the current study were 0.81 for the HADS-A and 0.72 for the HADS-D.

### Procedure

This study adhered to the Declaration of Helsinki of the World Medical Association and received approval from the Ethical Committee of Poznań University of Medical Sciences (KB-742/21). A longitudinal research design was implemented such that data were collected during two waves of a high COVID-19 infection rate in Poland (April 2021 and November 2021) and one wave of a low COVID-19 infection rate in Poland (July 2021). Initially, athletes involved in the Polish Paralympic Preparation Program were recruited for participation electronically and were sent an online survey link on the Google Forms platform in April 2021. Survey completion reminders were sent for a two-week period. Participants gave informed consent prior to completion of the survey. The survey included the questionnaire requesting demographic and sport-related information, the CISS, the pandemic-specific coping item, and HADS. The online surveys for which links were sent to participants in July 2021 and November 2021 both included the HADS.

### Data analysis

Descriptive statistics were calculated for the demographic- and disability-related variables, the CISS, and the HADS. A content analysis was performed on responses to the pandemic-specific coping item in which, after an initial examination of the data, a preliminary set of 10 general categories was identified, clarified through discussion between two investigators, and subsequently consolidated into six categories. Two independent raters then placed the open-ended responses into the categories and reached consensus. For participants who provided responses in two or more categories, only the first category identified was used in subsequent analyses.

To examine the extent to which differences in pandemic-specific coping in April 2021 were related prospectively to anxiety in July 2021 and November 2021, a mixed analysis of covariance (ANCOVA) with one within-subjects factor (i.e., time of assessment: July 2021 and November 2021), one between-subjects factor (i.e., pandemic-specific coping category), and six covariates (i.e., age, gender, April 2021 HADS-A scores, and CISS AO, EO, and TO scores) were performed on HADS-A scores. A parallel mixed ANCOVA was performed on HADS-D scores, substituting April 2021 HADS-D scores for April 2021 HADS-A scores as a covariate. An identical approach was taken for the longitudinal analyses comparing HADS scores in November 2021. A MANOVA was also conducted to compare April 2021 CISS AO, EO, and TO scores across pandemic-specific coping categories. Statistical analyses were conducted with IBM SPSS Statistics (Chicago, IL, United States) version 26.

## Results

Means and standard deviations of HADS-A and HADS-D scores for every pandemic-specific coping category are presented in Table 1. Responses to the pandemic-specific coping item tended to be brief, with a mean of 4.10 (*SD* = 4.72) words, a median of 2 words, and a mode of 1 word (*n* = 38, 42%). In the content analysis, six categories of responses to the pandemic-specific coping item were identified: cognitive coping (*n* = 44), social engagement (*n* = 17), no coping resources needed or listed (*n* = 11), maintaining composure/calmness (*n* = 9), distracting/engaging/sport behavior (*n* = 9), and other coping strategies (*n* = 1). Examples of cognitive coping included “optimism,” “positive attitude,” and “rational approach.” Examples of social engagement included “talking to beloved ones,” “conversation,” “family support,” and “contact with friends.” Examples of maintaining composure/calmness included “composure” and “calmness.” Examples of distracting/engaging/sport behavior include “hobby,” “reading books,” and “sport training.” The lone participant whose response was in the “other coping strategies” category was excluded from subsequent analyses.

**Table 1:** Means (and standard deviations) of HADS-A and HADS-D scores for pandemic-specific coping categories.

Variable		Pandemic-specific coping category				
		Cognitive coping(*n* = 44)	Composure/calmness(*n* = 9)	Distracting behavior(*n* = 9)	Social engagement(*n* = 17)	No coping needed or listed(*n* = 11)
HADS-A						
	April 2021	5.41 (3.81)	7.33 (3.67)	5.22 (3.87)	6.24 (3.11)	5.70 (3.65)
	July 2021	4.09 (3.50)	6.44 (6.04)	4.56 (3.32)	5.76 (2.93)	5.36 (3.72)
	Nov. 2021	4.93 (2.65)	5.78 (2.49)	5.11 (3.06)	6.29 (2.54)	7.18 (3.13)
HADS-D						
	April 2021	3.45 (2.59)	4.67 (3.24)	3.33 (3.57)	4.82 (3.91)	4.18 (4.31)
	July 2021	3.39 (2.95)	6.44 (5.74)	3.89 (3.33)	4.76 (3.87)	4.64 (4.13)
	Nov. 2021	4.05 (2.71)	5.00 (1.80)	6.33 (4.27)	6.00 (3.14)	6.45 (3.21)

Results of the ANCOVA comparing HADS-A scores of the five pandemic-specific coping categories across the July 2021 and November 2021 assessments are summarized in Table 2. Non-significant findings were obtained for the multivariate within-subjects tests of the time main effect (*p* = 0.79) and the interactions between time and gender (*p* = 0.38), AO coping (*p* = 0.93), EO coping (*p* = 0.19), TO coping (*p* = 0.35), and pandemic-specific coping (*p* = 0.52). Non-significant between-subjects effects were found for the age (*p* = 0.08), gender (*p* = 0.76), AO coping (*p* = 0.52), EO coping (*p* = 0.07), and TO coping (*p* = 0.92) covariates. Significant effects were obtained for the interaction between time and HADS-A scores (*p* = 0.007), the April 2021 HADS-A score covariate (*p* < 0.001), and the between-subjects effect of the pandemic-specific coping category (*p* = 0.048, partial-eta squared = 0.13). Paired comparisons revealed that participants in the no coping resources needed or listed category had significantly higher HADS-A scores than participants in the cognitive coping (*p* = 0.01) and maintaining composure/calmness (*p* = 0.03) categories across the July 2021 and November 2021 assessments.

**Table 2:** Summary of mixed ANCOVA for HADS-A scores.

	Wilks’ lambda	*F*	df	*p*	Partial eta-squared
Multivariate within-subjects tests					
Time	1.00	0.07	1,66	0.79	0.00
Time X age	1.00	0.10	1,66	0.76	0.00
Time X gender	0.99	0.79	1,66	0.38	0.01
Time X CISS-AO	1.00	0.01	1,66	0.93	0.00
Time X CISS-EO	0.97	1.77	1,66	0.19	0.03
Time X CISS-TO	0.99	0.89	1,66	0.35	0.01
Time X April 2021 HADS-A	0.89	7.87	1,66	0.007	0.11
Time X pandemic-specific coping category	0.95	0.81	1,66	0.52	0.05
Between-subjects tests					
Age		3.32	1,66	0.08	0.05
Gender		0.09	1,66	0.76	0.00
CISS-AO		0.41	1,66	0.52	0.01
CISS-EO		3.33	1,66	0.07	0.05
CISS-TO		0.01	1,66	0.92	0.00
April 2021 HADS-A		48.81	1,66	0.00	0.43
Pandemic-specific coping category		2.54	4,66	0.048	0.13

Results of the ANCOVA comparing HADS-D scores of the five pandemic-specific coping categories across the July 2021 and November 2021 assessments are summarized in Table 3. Non-significant findings were obtained for the multivariate within-subjects tests of the time main effect (*p* = 0.48) and the interactions between time and age (*p* = 0.43), AO coping (*p* = 0.93), EO coping (*p* = 0.19), TO coping (*p* = 0.35), and pandemic-specific coping (*p* = 0.06). Non-significant between-subjects effects were found for the age (*p* = 0.98), AO coping (*p* = 0.66), EO coping (*p* = 0.12), and TO coping (*p* = 0.42) covariates. Significant effects were obtained for the interaction between time and gender (*p* = 0.04), the interaction between time and April 2021 HADS-D scores (*p* = 0.004), the April 2021 HADS-D score covariate (*p* < 0.001), the gender (*p* = 0.02) covariate, and the between-subjects effect of the pandemic-specific coping category (*p* = 0.048, partial-eta squared = 0.12). Paired comparisons revealed that participants in the cognitive coping category had significantly lower HADS-D scores than participants in the no coping resources needed or listed (*p* = 0.01) and distracting/engaging/sport behavior (*p* = 0.03) categories across the July 2021 and November 2021 assessments. The MANOVA comparing April 2021 CISS AO, EO, and TO scores across pandemic-specific coping categories revealed a non-significant multivariate effect and no statistically significant univariate effects.

**Table 3:** Summary of mixed ANCOVA for HADS-D scores.

	Wilks’ lambda	*F*	df	*p*	Partial eta-squared
Multivariate within-subjects tests					
Time	1.00	0.50	1,79	0.48	0.01
Time X age	0.99	0.63	1,79	0.43	0.01
Time X gender	0.95	4.43	1,79	0.04	0.05
Time X CISS-AO	1.00	0.17	1,79	0.69	0.00
Time X CISS-EO	1.00	0.18	1,79	0.68	0.00
Time X CISS-TO	1.00	0.16	1,79	0.70	0.00
Time X April 2021 HADS-D	0.90	8.65	1,79	0.004	0.10
Time X pandemic-specific coping category	0.89	2.32	1,79	0.06	0.11
Between-subjects					
Age		0.00	1,79	0.98	0.00
Gender		5.39	1,79	0.02	0.06
CISS-AO		0.19	1,79	0.66	0.00
CISS-EO		2.54	1,79	0.12	0.03
CISS-TO		0.66	1,79	0.42	0.01
April 2021 HADS-D		70.95	1,79	0.00	0.48
Pandemic-specific coping category		2.54	4,79	0.048	0.12

## Discussion

In this study, the extent to which pandemic-specific coping was predictive of anxiety and depression in elite athletes with disabilities over and above general coping styles across multiple waves of the COVID-19 pandemic was investigated. Toward this aim, general coping styles were measured with a standardized self-report inventory and pandemic-specific coping was assessed with an open-ended item to capture unique efforts directed at dealing with stressors associated with the COVID-19 pandemic. Although analysis of participants’ open-ended responses resulted in coping categories that bore at least superficial resemblance to the AO, EO, and TO coping styles assessed with the CISS, the pandemic-specificity of the categories was supported by the lack of significant differences on the AO, EO, and TO subscales across the categories. Thus, participants who generally tended to engage in EO coping, for example, did not necessarily report using social engagement to deal with the COVID-19 pandemic. This finding aligns with the transactional model of stress and coping (Lazarus and Folkman, 1984) in that the novelty and unpredictability of the COVID-19 pandemic may have influenced cognitive appraisals of the situation and subsequent coping efforts in atypical ways.

General tendencies for AO, EO, and TO coping were significantly related to neither anxiety nor depression in the prospective, longitudinal analyses. These findings contrast with those of other studies in which general measures of coping were associated with mental health outcomes during the COVID-19 pandemic (Graupensperger et al., 2020; Leguizamo et al., 2021; Myall et al., 2021). Conversely, pandemic-specific coping was associated prospectively with both anxiety and depression when statistically controlling for age, gender, general coping styles, and the April 2021 values for anxiety and depression, respectively, during July 2021 and November 2021. The difference between the findings of the current study and those of previous studies may be attributable to the fact that although the participants in the investigations of Graupensperger et al. (2020), Leguizamo et al. (2021), and Myall et al. (2021) were high-level athletes, they were not athletes with disabilities. The COVID-19 pandemic may affect athletes with disabilities in unique ways that, consistent with the transactional model of stress and coping (Lazarus and Folkman, 1984), prompt coping efforts that diverge from the population of athletes in general (Puce et al., 2022).

The cross-group differences in anxiety were most pronounced between participants in the cognitive coping and maintaining composure/calmness groups and those in the no coping needed or listed group. These results speak to the potential long-term disadvantages of failing to deploy coping attempts and the advantages of cognitive coping and maintaining composure/calmness in the context of the COVID-19 pandemic. Participants in the cognitive coping and maintaining composure/calmness groups may have drawn upon their April 2021 coping experience to deal with pandemic-related stress in July and November, whereas participants in the no coping needed or listed group may have been unprepared to address pandemic-related stress in July and November 2021. As with anxiety, cognitive coping was associated with favorable outcomes for depression across the July 2021 and November 2021 assessments. Participants in the cognitive coping group reported lower levels of depression than those in the distracting/engaging/sport behavior and the no coping needed or listed groups. It is noteworthy that cognitive coping appeared not only to be an adaptive response to pandemic stress, but was also the most commonly indicated mode of dealing with the COVID-19 pandemic among the Polish athletes with disabilities in the current study. These findings align with the results of Fiorilli et al. (2021), which also pointed to the resilience and adaptability of disabled athletes during pandemic-related adversities. It is also important to note that coping effectiveness during this period might have been influenced by other factors not explored in these studies. Factors like motivation can drive engagement, including participation in physical activities and sports and the capability to overcome pandemic-related barriers (Han et al., 2021; Silva et al., 2022; Van Biesen and Morbee, 2023).

The elite Paralympic sample and prospective longitudinal assessments are clear strengths of the study, adding to the small body of research on coping and mental health in athletes with disabilities during the COVID-19 pandemic. Nevertheless, several limitations should be considered when interpreting the results. First, data were collected exclusively through self-report methods that are susceptible to the potential effects of forgetting and socially desirable responding, which may have affected the accuracy of the data. In future studies, observational methods and other approaches to data collection that do not rely solely on self-report should be considered to bolster confidence in the results. Second, the open-ended item used to assess pandemic-specific coping and the categorization scheme that was used to analyze responses to it have not been used previously and, therefore, have not been validated. Further inquiry is needed to determine the extent to which responses to the item reflect what respondents actually do to deal with the COVID-19 pandemic. Third, although the survey used in the study was sent to the entire population of participants in the Polish Paralympic Preparation Program, the sample size was smaller than would have been ideal for an investigation of this sort. Larger samples should be used in future research to increase both generalizability and statistical power. Fourth, given that participants were exclusively elite athletes with disabilities, the findings may not generalize to athletes with disabilities in general. Fifth, although a prospective longitudinal research design was used, three data collection episodes may not have been sufficient to adequately assess the full range of changes in coping and mental health over time. More frequent data collection episodes should be used in future research on the topic. Sixth, because a correlational research design was used in the current study, it is not possible to draw causal inferences from the findings. Experimental research designs should be used in future investigations to determine the extent to which various coping strategies influence the anxiety and depression of elite athletes with disabilities during a pandemic.

In addition to addressing the specific limitations of the current study, several more general suggestions for future research on the association between coping and mental health in the context of the COVID-19 pandemic are warranted. In line with the recommendations of Puce et al. (2022), it will be important for subsequent studies to engage the communities of interest in developing research questions and conducting the research, use psychometrically-sound instruments developed for the purpose and population under investigation, and implement multi-center, cross-national research designs.

## Conclusion

From an applied standpoint, the current findings suggest that during a pandemic, elite athletes with disabilities may use coping strategies that deviate from how they normally cope with more ordinary stressors, and that enlisting pandemic-specific coping resources may reap mental health rewards over the course of multiple waves of increased infection. Participants in the current study gravitated toward cognitive coping strategies, a choice made in April 2021 that was associated with low levels of anxiety 7 months later in November 2021. Future experimental research is needed to evaluate the relative merits of various coping strategies in producing adaptive mental health outcomes during a pandemic for elite athletes with disabilities. Such research would contribute to the development of interventions to assist elite athletes with disabilities to cope with pandemics and other atypical stressors.

## Data availability statement

The raw data supporting the conclusions of this article will be made available by the authors, without undue reservation.

## Ethics statement

This study was carried out in accordance with the Declaration of Helsinki of the World Medical Association and was approved by the Ethical Committee of Poznan University of Medical Sciences (KB-742/21). Written informed consent to participate in this study was provided by the patient/participants.

## Author contributions

PKU: Writing – original draft, Writing – review & editing, Conceptualization, Investigation. TT: Writing – review & editing. BWB: Writing – original draft, Writing – review & editing, Conceptualization, Investigation.
